# Dr. Dowler’s Physiological Researches

**Published:** 1852-01

**Authors:** 


### DR. DOWLER'S PHYSIOLOGICAL RESEARCHES.

Few American physicians have shown so resolute and persevering a spirit of research as Dr. Bennet Dowler, of New Orleans. For many years past he has been experimenting upon human bodies just after life had become extinct, and has elicited results of a very startling character. His observations as recorded amount to many thousand m. s. pages, and he is still pushing forward with the most praise-worthy zeal. Some of his earlier papers were published in our Journal, and he has since been an industrious contributor to several other journals.

We copy, under our head of selections, from the *New Orleans Medical and Surgical Journal*, a vindication of his claims to priority in certain views lately set forth by some French physiologists. We do this as an act of justice to our distinguished countryman, who is not only a man of true science and learning, but a gentleman of great amiability and moral worth. As has been the case in so many similar instances before, it would not be surprising if Dr. Dowler's discoveries had excited more interest everywhere else than at home, and should be more highly prized by future physiologists than they have been by his contemporaries.

